# Nandrolone Decanoate for Postmenopausal Osteoporosis: A Systematic Review and Meta-Analysis of Randomized Trials

**DOI:** 10.7759/cureus.98114

**Published:** 2025-11-29

**Authors:** Lucas C Camara, Matheus H Ferreira, Nelson C Junior

**Affiliations:** 1 Department of Clinical Anabolism, College of Governance, Engineering, and Education of SãoPaulo, São Paulo, BRA; 2 School of Physical Education, Physiotherapy, and Occupational Therapy, Universidade Federal de Minas Gerais (UFMG), Belo Horizonte, BRA; 3 Department of Sports Science and Biostatistics, Paulista University, São Paulo, BRA

**Keywords:** bone mineral density, lean mass, nandrolone decanoate, osteoporosis treatment, osteoporotic vertebral fracture, pain, postmenopausal women, systematic review and meta-analysis

## Abstract

Postmenopausal osteoporosis is often accompanied by reduced muscle mass and chronic bone pain, amplifying fracture risk and functional decline. Nandrolone decanoate (ND), a synthetic anabolic steroid, has been proposed as a dual-acting agent that may benefit both bone and muscle through osteoanabolic and myoanabolic mechanisms, yet its therapeutic value in osteoporosis remains uncertain. We conducted a systematic review and meta-analysis of randomized controlled trials comparing ND with placebo in postmenopausal women with primary osteoporosis. The review followed Preferred Reporting Items for Systematic Reviews and Meta-Analyses (PRISMA) 2020 guidelines and was prospectively registered in the International Prospective Register of Systematic Reviews (PROSPERO; CRD420251147647). Seven trials with 293 participants were included, with sample sizes varying by outcome. ND reduced fracture risk compared with placebo (moderate-certainty evidence). It produced modest increases in bone mineral density (BMD; low certainty) and more substantial gains in forearm bone mineral content (BMC; moderate certainty). ND also reduced pain (moderate certainty) and increased muscle mass (moderate certainty). However, ND was associated with a higher incidence of mostly mild virilizing adverse events (hirsutism, acne, voice changes; low certainty). Given the small sample sizes and methodological limitations of older trials, ND may be considered as a potential adjuvant option for selected postmenopausal women, particularly when muscle loss or refractory bone pain is present, provided treatment occurs under close clinical and laboratory monitoring. Larger, contemporary randomized trials are needed to define ND’s role within modern osteoporosis management.

## Introduction and background

Postmenopausal osteoporosis is a major public health issue associated with fragility fractures, pain, disability, and increased mortality [1,2]. Globally, over 200 million people are affected by osteoporosis, leading to approximately 8.9 million osteoporotic fractures each year [3]. Recent global meta-analytic estimates indicate a prevalence of 18.3%, affecting 23.1% of women and 11.7% of men [4]. These fractures contribute substantially to morbidity, healthcare costs, and loss of independence among older women, underscoring the urgent need for effective prevention and treatment strategies [3]. Despite the availability of established antiresorptive and anabolic therapies, several limitations persist. Oral bisphosphonates and denosumab are often associated with suboptimal adherence, potential rebound bone loss upon discontinuation, and rare but serious adverse events such as osteonecrosis of the jaw and atypical femoral fractures [5,6]. Likewise, anabolic agents such as teriparatide, abaloparatide, and romosozumab are limited by high cost, short treatment duration, and potential cardiovascular safety concerns [7,8]. Consequently, many women remain at high risk, particularly those with concomitant sarcopenia and chronic pain, highlighting the need for adjuvant strategies that act integratively on both bone and muscle [9,10]. This rationale is especially relevant in the context of osteosarcopenia, a condition in which the concomitant loss of bone and muscle markedly increases the risk of falls, fractures, and disability [9,11].

Nandrolone decanoate (ND) is a 19-nortestosterone derivative with anabolic and mild estrogenic properties via aromatization [12]. Despite its historical use in osteoporosis during the 1980s and 1990s, it fell out of favor due to androgenic concerns such as hirsutism and voice changes [13]. However, considering that a substantial proportion of patients with osteoporosis remain at elevated fracture risk despite standard antiresorptive therapy, particularly those with concomitant sarcopenia, re-evaluation of dual-acting agents targeting both bone and muscle is warranted. ND has demonstrated beneficial effects on bone mineral density (BMD) and lean mass, suggesting potential value as an adjuvant therapy in osteosarcopenia. Notably, ND remains explicitly licensed (on-label) for adjuvant treatment of postmenopausal osteoporosis in Brazil (Agência Nacional de Vigilância Sanitária (ANVISA)), Canada (Health Canada, historical monograph), and the United Kingdom (Medicines and Healthcare products Regulatory Agency (MHRA)), with approved dosing of 50 mg intramuscularly every three weeks to improve bone mineral density and reduce fracture risk [14-16]. In Europe, regulatory status varies by country, while in the United States (FDA), it is off-label for osteoporosis and approved only for anemia associated with chronic renal failure [17].

ND exerts complementary skeletal actions: it activates androgen receptors in osteoblasts and osteocytes and, partly through aromatization, modulates estrogenic pathways, thereby combining antiresorptive and osteoanabolic effects [18]. Its anabolic influence on muscle may contribute to improved strength and reduced fall risk, potentially translating into fewer fractures and establishing a physiological bone-muscle connection with clinical relevance in postmenopausal osteoporosis [11,18-20]. Nevertheless, ND has not yet been incorporated into most international osteoporosis guidelines [21,22], partly due to concerns regarding androgenic adverse events [13], as well as limitations of the existing evidence base, including small sample sizes, methodological constraints of early trials, and the scarcity of contemporary large-scale randomized studies.

To the best of our knowledge, no systematic reviews with meta-analyses of randomized clinical trials have, to date, synthesized the efficacy and safety of ND in postmenopausal women with osteoporosis. Therefore, the aim of this study is to conduct a systematic review and meta-analysis to comprehensively evaluate the effects of ND on efficacy and safety outcomes in this population. This synthesis aims to provide an updated and evidence-based perspective on ND’s therapeutic role in postmenopausal osteoporosis.

## Review

Methods

Study Objective and Registration

This study aimed to systematically evaluate the efficacy and safety of ND in postmenopausal women with primary osteoporosis. It was prospectively registered on the International Prospective Register of Systematic Reviews (PROSPERO) platform (CRD420251147647) on September 13, 2025, and conducted in accordance with Preferred Reporting Items for Systematic Reviews and Meta-Analyses (PRISMA) 2020 [23] and the Cochrane Handbook for Systematic Reviews of Interventions [24].

Eligibility Criteria

Types of studies: Randomized placebo-controlled clinical trials of ND as monotherapy in postmenopausal women with osteoporosis were included, with no language or publication status restrictions. ND monotherapy was selected to isolate its independent therapeutic effects and avoid confounding from combination regimens.

Participants: Postmenopausal women with a primary diagnosis of osteoporosis according to clinical, radiological, or densitometric criteria, as defined in each trial. Studies evaluating secondary osteoporosis (e.g., glucocorticoid-induced, renal, or oncologic) or participants with major comorbidities affecting bone metabolism were excluded.

Outcomes

Primary: Clinical fractures (vertebral and non-vertebral), as defined and adjudicated by each study using validated criteria when available.

Secondary: Changes in BMD at key sites (L1-L4, femoral neck, total hip), bone mineral content (BMC), pain (Visual Analog Scale (VAS)/Numeric Pain Rating Scale (NPRS)), muscle mass (prioritizing dual-energy X-ray absorptiometry (DXA) > MRI/CT > others), strength, functionality, quality of life, and adverse events (overall and androgenic).

The end of the intervention was used as the primary assessment time point, and key definitions and hierarchies are summarized in Appendices A, B.

Search and Selection

A comprehensive search was performed in Medical Literature Analysis and Retrieval System Online (MEDLINE), Excerpta Medica database (Embase), Cochrane Controlled Register of Trials (CENTRAL), and Latin America and the Caribbean Literature on Health Sciences (LILACS), as well as ClinicalTrials.gov and World Health Organization - International Clinical Trials Registry Platform (WHO-ICTRP), on September 16, 2025. The strategy combined MeSH terms such as “Osteoporosis, Postmenopausal”, “Osteoporosis”, and “Nandrolone”, along with related free-text keywords (full strategies in Appendix B). Grey literature sources (conference abstracts, theses, and preprints) were also screened. No language restrictions were applied. The last search update was conducted on November 16, 2025.

Two independent reviewers screened records in Rayyan (Rayyan Systems Inc., Cambridge, MA) [25], assessed eligibility, and extracted data. Disagreements were resolved by consensus or, when necessary, by a third reviewer. The study selection process is summarized in the PRISMA 2020 flow diagram [23].

Handling of Missing or Unclear Data

When essential data were missing or unclear, study authors were contacted for clarification. If unavailable, missing values were imputed from confidence intervals (CIs), standard errors, or interquartile ranges using standard Cochrane methods [24]. Sensitivity analyses were conducted to examine the influence of these imputations on pooled estimates, when necessary.

Risk of Bias

Risk of bias was independently assessed by two reviewers using the Cochrane Risk of Bias Tool 2.0 (RoB 2) tool across standard domains (randomization, deviations from intended interventions, missing data, measurement, and reporting). Each outcome was classified as low risk, some concerns, or high risk [26]. Discrepancies were resolved by consensus or consultation with a third reviewer.

Statistical Analysis

Analyses followed the Cochrane Handbook for Systematic Reviews of Interventions [24].

For dichotomous outcomes, risk ratios (RRs) were calculated; for continuous outcomes, mean difference (MD) or standardized mean difference (SMD) was used depending on scale comparability. Random-effects model (REML) and 95% CIs were applied.

Heterogeneity was evaluated via forest plots, Cochran’s Q test (p < 0.10), and I², interpreted per Cochrane thresholds (0%-40% = not important; 30%-60% = moderate; 50%-90% = substantial; 75%-100% = considerable).

Prespecified subgroup and sensitivity analyses aimed to explore differences according to ND dose, treatment duration, and follow-up length; these analyses were conducted when sufficient data were available. For adverse events, the primary analysis used the Mantel-Haenszel method, which applies continuity correction only to studies with 0 events in one arm. As a robustness check, we performed a sensitivity analysis applying a uniform continuity correction of 0.5 to all cells in all studies (incr = 0.5), allowing assessment of the impact of different correction strategies on the pooled effect. In meta-analyses with fewer than 10 studies, publication bias tests were interpreted cautiously; otherwise, funnel plots and Egger’s test were used [27].

All analyses were conducted in R (v4.5.1, The R Core Team, R Foundation for Statistical Computing, Vienna, Austria) [28] (via the metagen() and metabin() functions) [29] for meta-analyses and forest plots, dplyr and tidyr [30] for data cleaning and reshaping, and ggplot2 [31] for visualizations, including the risk of bias summary plot.

Certainty of Evidence

The certainty of the evidence for each outcome was assessed using the Grading of Recommendations, Assessment, Development, and Evaluation (GRADE) approach [32]. Two reviewers independently evaluated each outcome across five domains: risk of bias, inconsistency, indirectness, imprecision, and publication bias. Disagreements were resolved through consensus or consultation with a third reviewer.

Certainty was categorized as high, moderate, low, or very low and could be downgraded based on methodological concerns or upgraded for large effects, dose-response gradients, or residual confounding. All judgments and justifications were summarized in a Summary of Findings (SoF) table generated using GRADEpro (Evidence Prime, Hamilton, Ontario, Canada).

Results

The process of study identification, screening, eligibility, and inclusion is presented in the PRISMA 2020 flow diagram [23]. A total of 217 records were identified in the databases, with no additional records from clinical trial registries or other sources. After removing 22 duplicates, 195 records remained for title and abstract screening, of which 179 were excluded for not meeting eligibility criteria.

**Figure 1:**
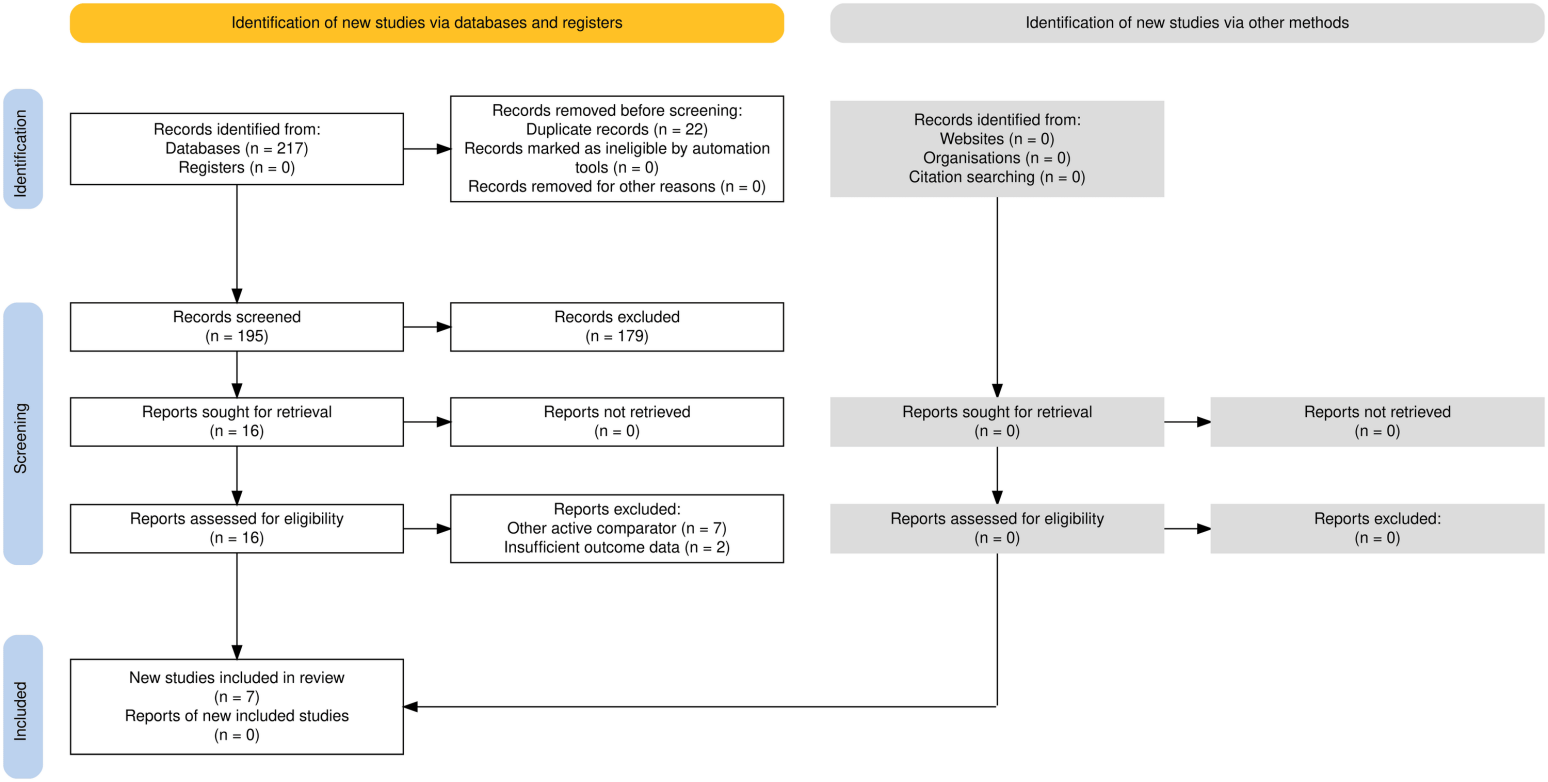
A PRISMA 2020 flow diagram summarizing the study selection process, including identification, screening, eligibility assessment, and final inclusion PRISMA: Preferred Reporting Items for Systematic Reviews and Meta-Analyses [23]

Sixteen studies were assessed in full text. Of these, seven were excluded for using active comparators and two for presenting insufficient outcome data. Thus, seven studies met all inclusion criteria and were considered in the qualitative and quantitative synthesis. This process ensures transparency and reproducibility of the review, as recommended by the PRISMA 2020 statement (Figure 1) [23].

The main characteristics of the randomized clinical trials included are summarized in Table 1. Most studies were conducted in Europe during the 1980s and 1990s, with one more recent trial conducted in Brazil in 2005. All evaluated postmenopausal women with osteoporosis diagnosed by clinical, radiological, or densitometric criteria and administered ND intramuscularly, generally at a dose of 50 mg every three weeks. Comparators were placebos, with or without calcium supplementation, and follow-up periods ranged from six to 24 months. Although the outcomes assessed varied, most studies reported measures of BMD or BMC, body composition, and fractures, allowing for quantitative synthesis.

**Table 1:** Characteristics of randomized controlled trials included in the review ND: nandrolone decanoate; Ca: calcium; IM: intramuscular; BMD: bone mineral density; BMC: bone mineral content; ALP: serum alkaline phosphatase; Hb: hemoglobin; AST: aspartate aminotransferase; DBP: vitamin D–binding protein; VAS: visual analog scale for pain; FMC: forearm mineral content; UNIFESP: Federal University of São Paulo

Study (year)	Country/setting	N randomized (ND/Placebo)	Population (age; T-score)	ND regimen (route, dose, frequency)	Comparator/co-interventions	Follow-up (months)	Outcomes assessed	Notes
Gennari et al., 1989 [33]	Italy (University of Siena)	20 (10/10)	Postmenopausal women with ≥1 vertebral fracture; lumbar BMC < –2 SD	IM, 50 mg, every 3 weeks	Placebo + 1 g Ca/day	12	Lumbar and femoral BMC; ALP; urinary hydroxyproline; intestinal Ca absorption; bone biopsy	Double-blind, placebo-controlled
Hassager et al., 1989 [34]	Denmark (Glostrup Hospital)	39 (20/19)	Postmenopausal osteoporotic women (prior spine or Colles fracture)	IM, 50 mg, every 3 weeks	Placebo + 500 mg Ca/day	12	Body composition (lean/fat mass); lipid profile; urinary creatinine; hemoglobin; AST	Double-blind, placebo-controlled
Johansen et al., 1989 [35]	Denmark (Glostrup Hospital)	39 (20/19)	Women 55–75 years, postmenopausal, ≥1 osteoporotic fracture (spine/forearm)	IM, 50 mg, every 3 weeks	Placebo + 500 mg Ca/day	12	Forearm BMC (proximal/distal); lumbar BMD; bone-formation markers; 99mTc-DP retention	Double-blind, controlled
Hartwell et al., 1990 [36]	Denmark (Glostrup Hospital)	39 (19/20)	Women 55–75 years, postmenopausal, ≥1 osteoporotic fracture (spine/forearm)	IM, 50 mg, every 3 weeks	Placebo + 500 mg Ca/day	12	Vitamin D metabolism (1,25(OH)₂D, DBP, 24-hydroxylase); urinary Ca, phosphate and cAMP excretion	Double-blind, placebo-controlled
Need et al., 1993 [37]	Australia (Adelaide)	45 (crossover)	Women with forearm density <95% of young normal; no vertebral fractures	IM, 50 mg, every 4 weeks	Placebo	6 (per period)	Forearm mineral content (FMC); L2–L4 BMD; creatinine; hemoglobin; forearm fat; phosphate; ALP; HDL	Double-blind, crossover
Passeri et al., 1993 [19]	Italy (University of Parma)	46 (25/21)	Women 46–68 years, postmenopausal, ≥1 vertebral compression fracture	IM, 50 mg, every 3 weeks	Placebo + 1 g Ca/day	18	Lumbar and distal radius BMD; bone turnover (osteocalcin, hydroxyproline); pain (VAS); vertebral fractures; Hb; HDL	Double-blind, placebo-controlled
Frisoli et al., 2005 [20]	Brazil (UNIFESP)	65 (32/33)	Women >70 years, osteoporotic; T-score ≤ –2.5 SD	IM, 50 mg, every 3 weeks	Placebo + 500 mg Ca/day	24	Vertebral fracture; lumbar/neck/trochanter BMD; muscle mass; hemoglobin	Double-blind, placebo-controlled

Risk of Bias

**Figure 2:**
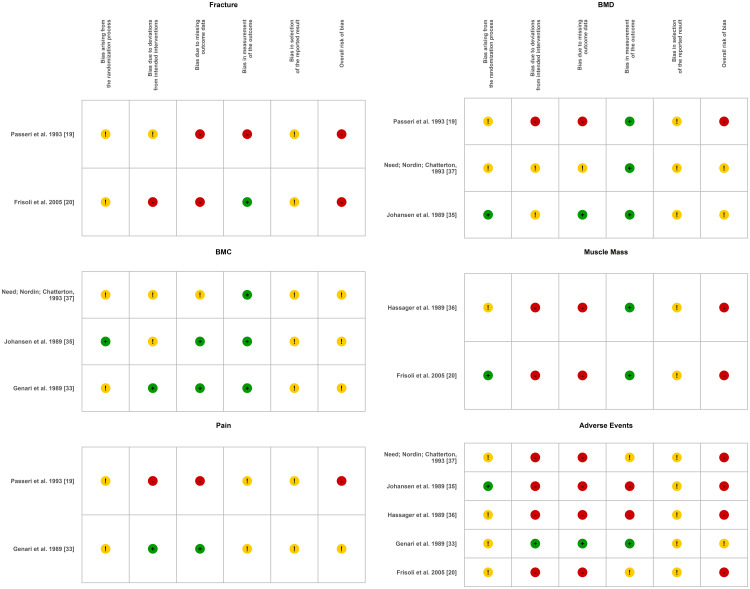
Risk of bias assessment for each outcome using the Cochrane Risk of Bias Tool 2.0 (RoB 2) tool Color codes represent the risk of bias assessment: Green, low risk; Yellow, some concerns; Red, high risk [26]. BMD: bone mineral density; BMC: bone mineral content

Risk of bias was assessed using RoB 2 for each outcome, strictly following the decision algorithms [24,26]. No manual overrides were applied, and “No information” responses were preserved. Final judgments were categorized as low risk, some concerns, or high risk. The proportions by domain and outcome are presented in Figure 2.

In summary, fractures and adverse events showed the highest proportion of high-risk classifications. BMC outcomes consistently exhibited some concerns, whereas BMD, lean mass, and pain demonstrated more heterogeneous risk profiles. These findings were incorporated into the sensitivity analyses and into the assessment of certainty of evidence using the GRADE approach [32].

A considerable portion of high-risk ratings stemmed from incomplete reporting in older trials (1980s-1990s), particularly missing details on random sequence generation and allocation concealment, rather than from confirmed methodological flaws. Because RoB 2 was applied strictly by outcome, subjective outcomes (e.g., pain) were more susceptible to performance and detection bias due to limited blinding, whereas objective outcomes (e.g., BMC) tended to yield more balanced risk assessments. These considerations were incorporated when interpreting the overall certainty of evidence using the GRADE approach [32].

Bone Fractures

**Figure 3:**
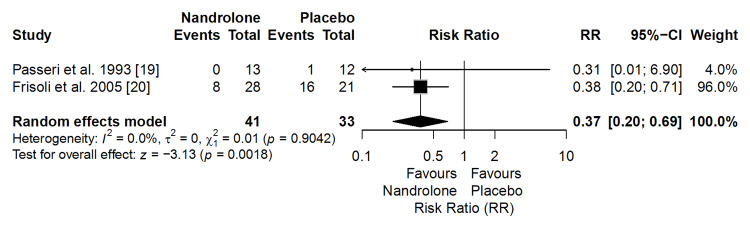
Forest plot of the meta-analysis on fracture risk (effect measure: risk ratio (RR))

The meta-analysis of two clinical trials (n = 74) showed that ND significantly reduced the risk of fractures compared with placebo (RR = 0.37; 95% CI: 0.20 to 0.69; p = 0.0018), with no evidence of heterogeneity (I² = 0%). This corresponds to a 63% relative reduction in fracture risk (Figure 3).

According to the GRADE approach, the certainty of the evidence was rated as moderate due to imprecision, despite low heterogeneity and a statistically significant effect.

BMD

**Figure 4:**
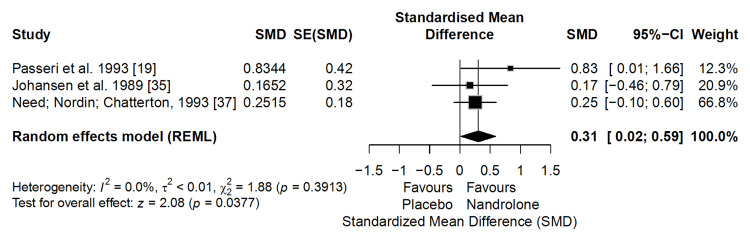
Forest plot of the meta-analysis on bone mineral density (BMD) (effect measure: standardized mean difference (SMD))

The meta-analysis of three clinical trials (n = 92) showed that ND significantly increased BMD compared with placebo (SMD = 0.31; 95% CI: 0.02 to 0.59; p = 0.0377), with no evidence of heterogeneity (I² = 0%) (Figure 4). This SMD corresponds to an absolute gain of approximately 0.03-0.05 g/cm², assuming a typical BMD standard deviation of 0.10-0.15 g/cm² in postmenopausal women. In epidemiological cohorts, each 1 SD increase in BMD is associated with a 40%-50% reduction in vertebral fracture risk and a 30%-40% reduction in hip fracture risk [38,39]. Although the observed increase is smaller than 1 SD, it still represents a clinically meaningful improvement in BMD.

According to the GRADE approach, the certainty of the evidence was rated as low due to a serious risk of bias and inconsistency.

BMC

**Figure 5:**
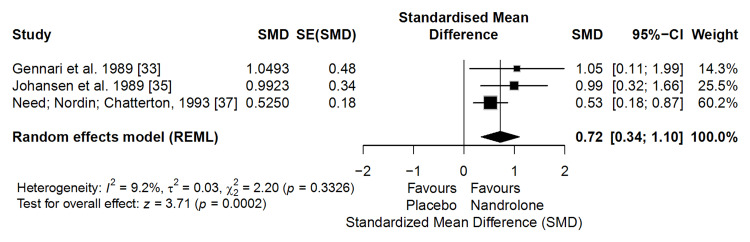
Forest plot of the meta-analysis on bone mineral content (BMC) (effect measure: standardized mean difference (SMD))

The meta-analysis of three clinical trials (n = 96) showed that ND significantly increased BMC compared with placebo (SMD = 0.72; 95% CI: 0.34-1.10; p = 0.0002), with low heterogeneity (I² = 9.2%) (Figure 5). This SMD corresponds to an approximate absolute gain of 2.5-4.4%, assuming a typical BMC standard deviation of 3.5%-6.0% in postmenopausal women. Given that untreated postmenopausal women typically lose 1%-2% of BMC annually [40,41], such gains represent a clinically relevant anabolic response, comparable to the ~3% increase in total-body BMC achieved with teriparatide, an established anabolic agent [41].

According to the GRADE approach, the certainty of evidence for this outcome was rated as moderate, primarily due to some concerns regarding risk of bias in older trials but supported by consistent effect estimates, low heterogeneity, and precise confidence intervals.

Pain (VAS)

**Figure 6:**
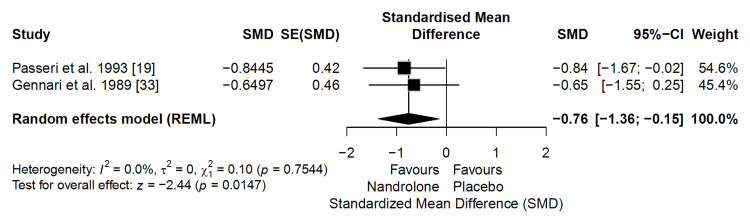
Forest plot of the meta-analysis on pain (Visual Analogue Scale) (effect measure: standardized mean difference (SMD)) Reference: [44]

Based on two clinical trials (n = 45), ND significantly reduced pain compared with placebo (SMD = −0.76; 95% CI: −1.36 to −0.15; p = 0.0147), with no evidence of heterogeneity (I² = 0%) (Figure 6). This corresponds to an absolute reduction of approximately 1.5-1.9 points on the VAS (assuming a typical SD of 2.0-2.5), exceeding the minimal clinically important difference of 1.3-2.0 points [42,43]. Both studies used comparable pain assessment scales, ensuring consistency in the outcome. The certainty of evidence was rated as moderate using the GRADE approach, suggesting that ND probably reduces pain in postmenopausal women with osteoporosis.

Muscle Mass

**Figure 7:**
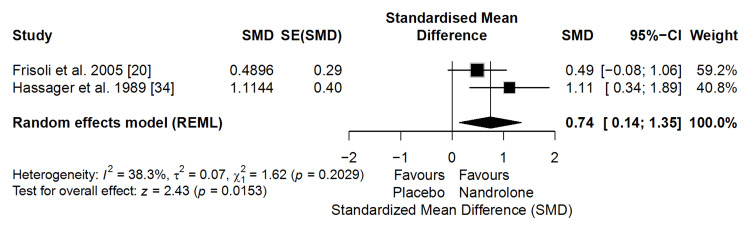
Forest plot of the meta-analysis on muscle mass (effect measure: standardized mean difference (SMD))

The meta-analysis of two clinical trials (n = 82) showed that ND significantly increased muscle mass compared with placebo (SMD = 0.74; 95% CI: 0.14 to 1.35; p = 0.0153). Despite non-significant heterogeneity (I² = 38.3%), the consistency in the direction of effects across studies strengthens the confidence in the observed association (Figure 7). This SMD corresponds to an estimated absolute gain of approximately 1.2 to 1.8 kg of muscle mass, assuming typical standard deviations reported in postmenopausal women. Gains of this magnitude exceed thresholds associated with improvements in strength, mobility, and physical function [45]. Overall, these findings indicate a clinically meaningful anabolic effect of ND on muscle mass.

According to the GRADE framework, the certainty of evidence for this outcome was rated as moderate. This rating reflects some concerns related to risk of bias in older trials, while being supported by consistent direction of effect, acceptable precision, and absence of important heterogeneity.

Adverse Events

**Figure 8:**
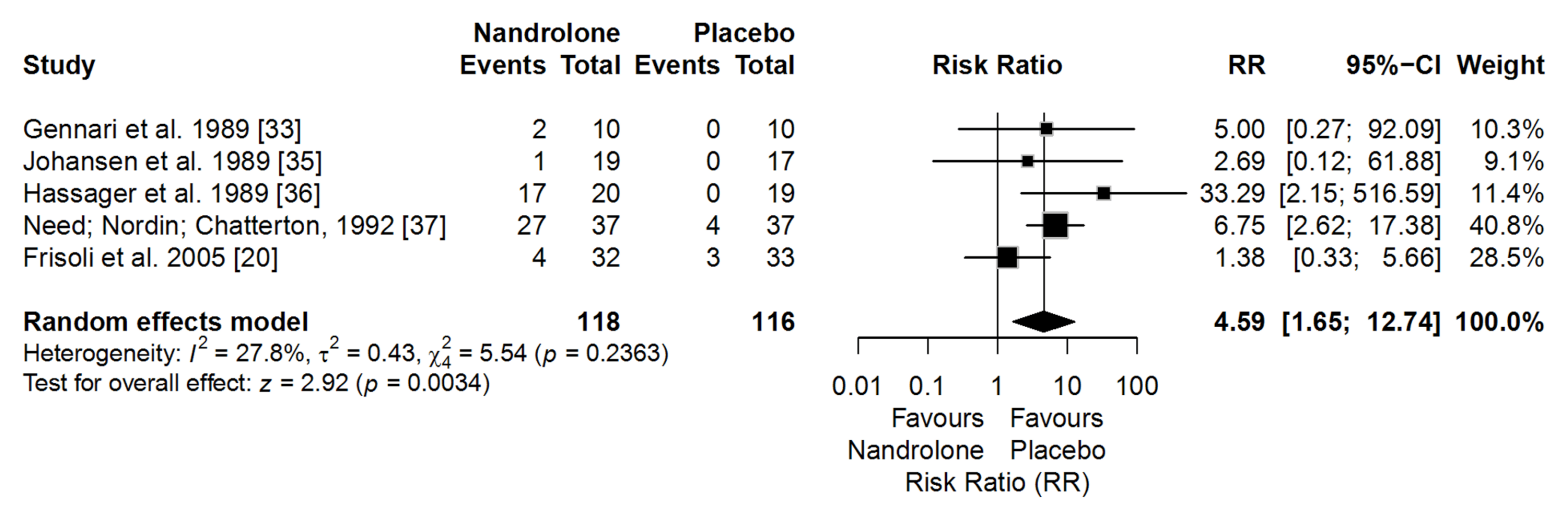
Forest plot of the meta-analysis on adverse events (effect measure: risk ratio (RR))

Based on five clinical trials (n = 234), the use of ND was associated with a significantly higher risk of adverse events compared with placebo (RR = 4.59; 95% CI: 1.65-12.74; p = 0.0034). Heterogeneity was low (I² = 27.8%) (Figure 8). 

In absolute terms, adverse events occurred in approximately 15.8% of participants receiving ND and 3.5% of those receiving placebo, reflecting an absolute risk difference of 12.3 percentage points.

According to the GRADE approach, the certainty of evidence for this outcome was rated as low. This rating reflects concerns regarding risk of bias, largely due to incomplete reporting of adverse event monitoring in older trials, and imprecision driven by low event counts, despite the consistent direction of effect and low heterogeneity.

The sensitivity analysis applying a uniform continuity correction (adding 0.5 to all cells) yielded an RR of 4.28 (CI: 1.54; 11.87; p = 0.0052), maintaining both the significance and direction of the effect.

Reported androgenic effects, including mild to moderate hirsutism, transient hair loss, and vocal changes (hoarseness or deepening), were generally reversible after treatment discontinuation. No serious or life-threatening events were reported.

**Figure 9:**
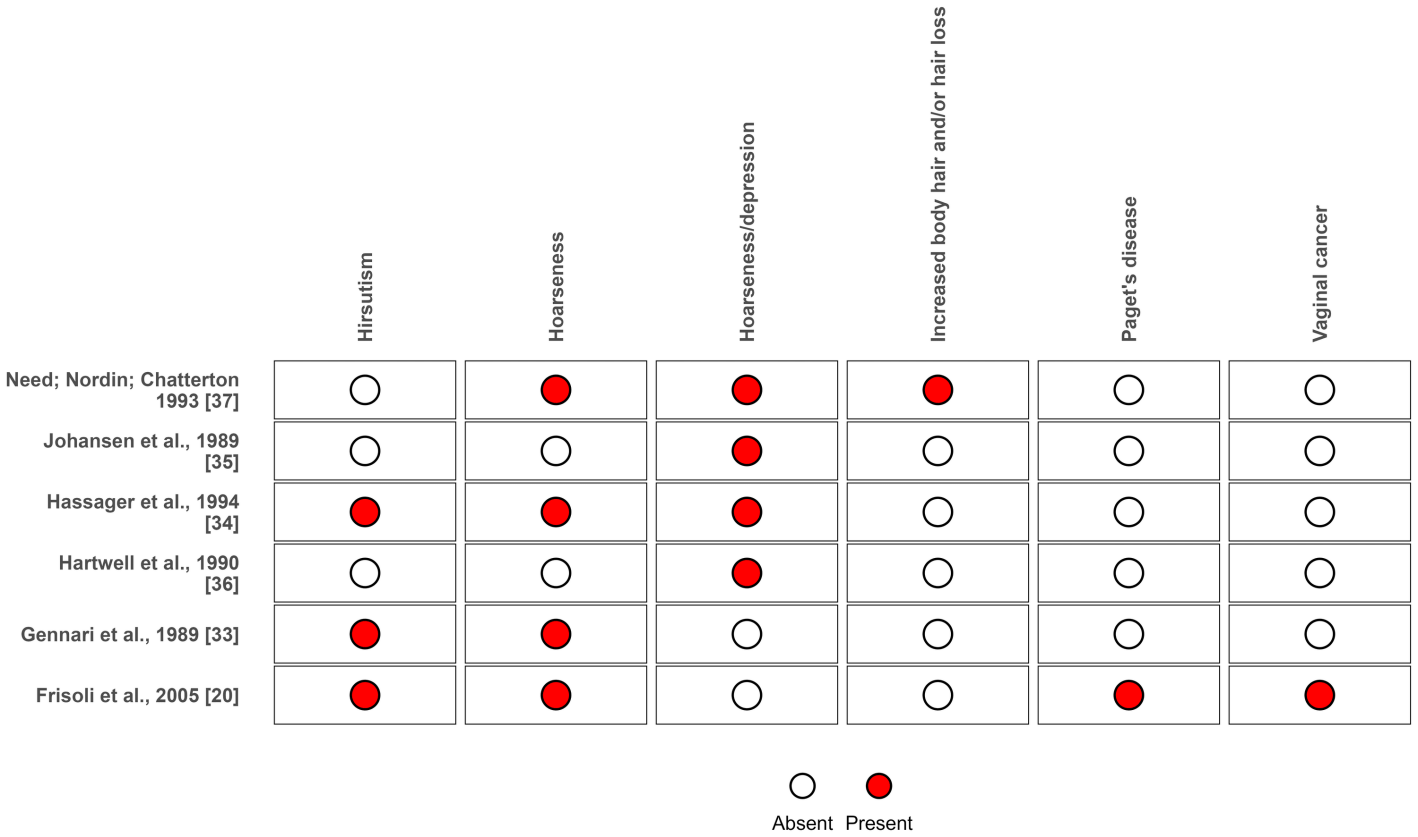
Adverse events reported in individual randomized clinical trials of nandrolone decanoate

The main adverse events observed in each study are listed in Figure 9.

Muscle Strength, Functional Capacity, and Quality of Life

For the outcomes of muscle strength, functional capacity, and quality of life, meta-analyses could not be performed because only one randomized clinical trial assessed each of these outcomes individually. Therefore, the available results were described narratively, with no possibility of quantitative synthesis.

Certainty of Evidence: GRADE

To synthesize the strength and reliability of the findings, we applied the GRADE approach [32]. This tool allows the certainty of the evidence to be classified by considering risk of bias, inconsistency, imprecision, publication bias, and indirectness. Appendix C presents the GRADE assessment for the main outcomes, including the absolute and relative effect estimates and the confidence level assigned to each. This summary facilitates the clinical interpretation of the results and highlights the areas where the evidence remains uncertain or limited.

Discussion

In this systematic review with meta-analysis, ND was shown to reduce the risk of fractures (moderate-certainty evidence), increase BMD (low-certainty evidence) and BMC (moderate-certainty evidence), reduce pain (moderate-certainty evidence), increase muscle mass (moderate-certainty evidence), and, in contrast, increase the occurrence of adverse events (low-certainty evidence). To the best of our knowledge, this is the first study to comprehensively evaluate all of these clinically relevant outcomes, determining not only the magnitude of the effects but also the certainty of the evidence according to the GRADE approach [32]. These findings provide a broad perspective on the potential and limitations of nandrolone use in the management of postmenopausal osteoporosis. 

Our findings regarding fractures are consistent with classic articles in the literature. Dequeker & Geusens (1986) [46] demonstrated a consistent protective effect of ND against fractures when compared with 1α-hydroxycholecalciferol, sustained even after discontinuation. Complementarily, a recent systematic review highlighted that ND improves lean mass and bone parameters, emphasizing evidence of fracture benefit from historical clinical trials [47]. Convergently, Dave et al. (2025) [13], in a prospective observational study of 100 postmenopausal women, reported significant improvement in BMD and functional outcomes, with no incident fractures recorded during follow-up, suggesting an indirect protective effect. In addition, narrative reviews have discussed the mechanistic plausibility that ND, by combining osteoanabolic action with increases in muscle mass, contributes to fracture risk reduction, reinforcing the clinical potential of this drug in the context of osteoporosis and osteosarcopenia [48].

With regard to BMD and BMC, studies not included in the present review that used other drugs as control groups also reported benefits with nandrolone for these outcomes. In summary, the evidence is more consistent for lumbar spine and vertebral BMD [49,50], with more pronounced effects in trabecular bone (lumbar region) than in cortical bone (forearm) [49]. In two studies, the nandrolone-treated group showed a significant increase in BMD compared with both baseline values and the control group [49,50]. Additionally, Szücs et al. (1992) demonstrated maintenance of bone mass for up to three years when ND was combined with calcitonin, in contrast to the progressive bone loss observed in comparator groups without nandrolone [51]. The mechanistic plausibility, the combination of osteoanabolic and antiresorptive action through androgen receptors, and the gain in muscle mass that may reduce falls are consistent with the outcomes observed [20,48,49].

However, in a study not included in the present meta-analysis because it used hormone replacement therapy (HRT) as the control group, no differences were found between groups for the forearm BMC outcome [49]. In that trial, both HRT alone and HRT combined with ND resulted in significant increases in BMC and BMD compared with baseline, but the additional effect of ND did not reach statistical significance. This result may be explained by the small sample size (n = 36 at the end of follow-up), the high efficacy of HRT itself [52-54], which had already produced substantial gains in bone mass, and the relatively short follow-up period to detect incremental differences between active therapies.

With regard to the gain in muscle mass with ND use demonstrated in the present meta-analysis, the literature consistently supports this effect. Clinical trials such as those by Hassager et al. (1989) [34], Frisoli et al. (2005) [20], and Dave et al. (2023) [11] reported significant increases in this outcome, in some cases accompanied by functional improvement. These findings were corroborated by systematic reviews, such as that of Câmara et al. (2023) [47]. In our analysis, the certainty of the evidence was rated as moderate, which further strengthens this result. The mechanistic plausibility that androgens act directly on muscle, promoting hypertrophy and improved functional performance, is also widely described [48].

In addition to the impact on BMD and BMC, our findings on pain reduction are consistent with the literature. Passeri et al. (1993) [19] observed relevant symptomatic improvement in osteoporotic women treated with ND, while Gennari et al. (1989) [33], in a double-blind placebo-controlled trial, documented a significant reduction in pain early in treatment, with the benefit maintained throughout the 12-month follow-up. Convergently, a comprehensive narrative review reinforced that ND reduces vertebral pain and improves functional mobility, consolidating the clinical plausibility of this benefit [55].

In the clinical trials evaluated, adverse events associated with ND use were relatively frequent, generally affecting more than 30% of participants, but were predominantly mild to moderate in intensity. The most commonly reported effects included acne, hirsutism, hair loss, and vocal changes, while menstrual alterations (in older studies) and mild laboratory abnormalities in hemoglobin or lipid profiles were also observed [20,37]. Serious adverse events were rare, and no life-threatening cases were reported. The incidence of adverse events was higher than with placebo and showed a dose-dependent relationship [20,37].

From a clinical perspective, these findings suggest that although ND provides clinically meaningful improvements in bone density, muscle mass, and fracture outcomes, its use should be accompanied by careful patient selection and regular monitoring for androgenic effects, particularly in long-term therapy. The overall risk-benefit profile supports ND as a potential adjuvant therapy when conventional options are insufficient or poorly tolerated, provided that appropriate surveillance and dose control are maintained.

We observed a higher proportion of “high risk” judgments among older trials. This pattern is consistent with historical reporting limitations prior to the consolidation of CONSORT 2010 in aspects such as sequence generation/concealment, blinding, intention-to-treat analysis, and public registration/protocol [56]. These gaps affect core domains of RoB 2 (D1-D5) and, in combination, lead the algorithm to classify results as “high risk.” Moreover, meta-epidemiological studies show that inadequate/absent allocation concealment and lack of blinding tend to overestimate effects and increase heterogeneity, particularly in subjective outcomes [57-59]. Therefore, retaining the “high risk” judgments indicated by the algorithm is methodologically justified.

Taken together, the findings indicate a benefit-risk profile of ND that should be assessed individually. The potential clinical benefits in fracture risk, pain, BMD/BMC, and muscle mass [13,19,20,33,34,47,50] are counterbalanced by a higher incidence of adverse events, mostly of mild to moderate intensity [20,35,37,55]. The decision to use ND should consider the severity of fracture risk, the concomitant presence of sarcopenia, failure or intolerance to standard therapies, and patient preferences.

The applicability of the evidence is clearer in postmenopausal women with established osteoporosis [19,55], particularly in those with markedly reduced lean mass [20,34] or refractory pain [33]. In addition to modern anabolic therapies such as teriparatide, abaloparatide, and romosozumab, which have demonstrated fracture reduction versus placebo and increases in BMD in high-risk patients, our findings suggest that ND, although less explored in current guidelines, also exerts a clinically relevant osteoanabolic effect. In a prospective study, the addition of ND to alendronate promoted further gains in lumbar BMD and improvements in function and quality of life [18]. Thus, ND emerges as a candidate to be tested in specific scenarios, although direct comparisons with modern anabolic agents remain a research gap.

According to current meta-analyses and guidelines, bisphosphonates remain the first-line therapy for reducing vertebral and non-vertebral fractures, while denosumab and teriparatide (or other anabolic agents) are recommended for very high-risk cases or in cases of intolerance/failure to bisphosphonates. In this context, ND may complement standard therapy in subgroups where the component of lean mass loss (osteosarcopenia) and/or persistent pain is decisive, as it combines osteo (anabolic/antiresorptive) effects with the muscle anabolism observed in this review. To confirm incremental benefit and safety compared with active comparators, head-to-head trials with clinical outcomes (vertebral and non-vertebral fractures), as well as functional measures and quality of life, are needed [60-68].

The heterogeneity across studies, particularly in the therapeutic regimen used (50 mg intramuscular every three weeks versus monthly) and in duration (12 to 36 months), likely contributes to the variation in effect observed between trabecular sites, such as the lumbar spine, and cortical sites, such as the radius and forearm [55,69]. In the future, trials should standardize doses, adopt minimum follow-up windows of ≥24 months consistent with recommendations for assessing fracture outcomes [70], and investigate dose-response gradients, including tapering and maintenance strategies.

From a practical standpoint, given the increase in adverse events, a minimum monitoring protocol is required. This includes baseline assessment with complete blood count, liver enzymes, lipid profile, blood pressure, and history of androgenic signs; reassessment at six to eight weeks and then quarterly during the first semester, followed by semiannual evaluations thereafter; clinical surveillance for acne, hirsutism, hoarseness, and menstrual alterations, along with continuous laboratory monitoring; and explicit criteria for discontinuation in cases of intolerable adverse events or laboratory complications.

Multicenter clinical trials with larger samples, rigorous blinding, and follow-up of at least two years are a priority. These studies should use modern active comparators and evaluate clinically relevant outcomes, such as vertebral and non-vertebral fractures, as well as standardized outcomes of body composition, physical function, and quality of life. They should also include specific subgroups, such as very elderly women or those with osteosarcopenia, bone remodeling biomarkers, cost-effectiveness analyses, and prospective pharmacovigilance strategies.

In summary, ND has been shown to reduce the risk of fractures and pain, increase BMD/BMC, and substantially raise lean mass, although it is associated with a higher incidence of clinically mild adverse events compared with placebo. In selected clinical contexts and under appropriate monitoring, it may represent a promising adjuvant option. However, additional confirmatory trials with greater methodological rigor are essential to ensure the sustained safety and efficacy of its clinical use.

The findings suggest applicability primarily in postmenopausal women with established osteoporosis who are refractory or intolerant to standard therapies, particularly when sarcopenia and/or refractory pain are present. In these scenarios, ND may complement care by combining osteoanabolic and antiresorptive actions with muscle anabolism, potentially reducing fall and fracture risk. Its use should occur under structured monitoring and after shared decision-making regarding benefits and risks.

Nevertheless, ND’s precise role within current osteoporosis treatment strategies remains to be defined, as evidence is still limited regarding direct comparisons with modern anabolic agents, standardized dosing regimens, and the need for contemporary long-term data under modern safety monitoring standards.

Limitations

The limitations of this study are as follows: small sample sizes limit the precision and generalizability of pooled estimates; short follow-up durations restrict the ability to evaluate long-term efficacy and sustained safety; the high proportion of “high risk of bias” ratings reflects incomplete reporting in older trials rather than confirmed methodological flaws; outcome-level RoB judgments prioritized clinically relevant endpoints, and complementary publications were consulted when available; heterogeneity in dosing regimens, treatment durations, and outcome measures complicates meta-analytic pooling and may contribute to residual variability; Older densitometry techniques reduce the comparability of BMD and BMC across studies; limited blinding information in several trials may have inflated subjective outcomes; only two trials reported fracture outcomes, introducing imprecision and limiting the strength of evidence for this endpoint; economic, subgroup, and cost-effectiveness data were unavailable, constraining clinical and policy-level applicability.

## Conclusions

ND appears to reduce fracture incidence (moderate-certainty evidence), alleviate pain (moderate certainty), increase BMD (low certainty), and enhance BMC (moderate certainty), while improving muscle mass (moderate certainty) and increasing primarily mild androgenic adverse events (low certainty).

Considering these findings, and acknowledging the limitations of small, older trials with relatively short follow-up, ND may be considered a potential adjuvant therapy for selected postmenopausal women, provided that treatment occurs under rigorous clinical and laboratory monitoring. Although ND remains approved as an adjuvant therapy for postmenopausal osteoporosis in several countries, its precise role in contemporary osteoporosis management remains uncertain. Larger, modern randomized controlled trials comparing ND with established anabolic and antiresorptive agents (e.g., romosozumab, abaloparatide) are needed to clarify its therapeutic value and confirm these results.

## Appendices

Appendix A

Database Search Strategies

**Table 2:** Complete search strategies for all databases included in the systematic review

Database	Search strategy (filters and Boolean operators)
LILACS (Filters applied: randomized controlled trial)	MH:"Osteoporose Pós-Menopausa" OR MH:"Osteoporosis Posmenopáusica" OR MH:"Osteoporosis, Postmenopausal" OR (postmenopausal osteoporosis) OR (Postmenopausal bone loss) OR (perimenopausal bone loss) OR (bone loss postmenopausal) OR (osteoporosis post-menopausal) OR (osteoporosis postmenopáusica) OR (pérdida ósea posmenopáusica) OR (perda óssea pos-menopausa) OR MH:C05.116.198.579.610$ OR MH:C18.452.104.579.610$ AND MH:Nandrolona OR MH:Nandrolone OR (19-Nortestosterona) OR Estrenolona OR Norandrostenolona OR Nortestosterona OR Nandrolone OR (17-Hydroxy-Estr-4-Ene-3-One) OR (17beta-Hydroxy-19-Nor-4-Androsten-3-One) OR (19-Nortestosterone) OR Norandrostenolone OR mh:D04.210.500.365.415.638$ OR mh:D06.472.334.851.968.976$
PubMed / MEDLINE	(("Osteoporosis"[Mesh] OR Osteoporoses OR (Osteoporosis, Post-Traumatic) OR (Osteoporosis, Senile) OR (Osteoporosis, Age-Related) OR (Bone Loss, Age-Related)) OR ("Osteoporosis, Postmenopausal"[Mesh] OR (Perimenopausal Bone Loss) OR (Postmenopausal Bone Loss) OR (Bone Losses, Postmenopausal))) AND ("Nandrolone"[Mesh] OR (19-Nortestosterone) OR (17beta-Hydroxy-19-Nor-4-Androsten-3-One) OR Estrenolone OR Nortestosterone OR Norandrostenolone OR Retabolil OR Retabolyl OR Decadurabolin)) AND ((randomized controlled trial[pt] OR controlled clinical trial[pt] OR randomized[tiab] OR placebo[tiab] OR drug therapy[sh] OR randomly[tiab] OR trial[tiab] OR groups[tiab]) NOT (animals [mh] NOT humans [mh]))
CENTRAL (Cochrane Library)	#1 MeSH descriptor: [Osteoporosis, Postmenopausal] explode all trees; #2 Osteoporosis OR (Post-Menopausal Osteoporosis) OR (Bone Loss, Postmenopausal); #3 #1 OR #2; #4 MeSH descriptor: [Nandrolone Decanoate] explode all trees; #5 Retabolil OR Retabolyl OR Decadurabolin OR (19 Nortestosterone Decanoate); #6 #4 OR #5; #7 MeSH descriptor: [Nandrolone] explode all trees; #8 (19 Nortestosterone) OR (17beta Hydroxy 19 Nor 4 Androsten 3 One) OR Estrenolone OR Norandrostenolone; #9 #7 OR #8; #10 #3 AND #6 AND #9
TRIP Database	(title:(postmenopausal osteoporosis) OR (osteoporosis)) AND (title:(nandrolone decanoate) OR (nandrolone))
SciELO	nandrolone OR "nandrolone decanoate" OR norandrostenolone OR retabolyl OR esterolone OR decadurabolin OR "nortestosterone decanoate" OR retabolil OR decadurobolin OR "19-nortestosterone"
OpenGrey	Nandrolone OR Nandrolone Decanoate OR Nortestosterone OR Retabolil OR Retabolyl OR Decadurobolin OR Decadurabolin OR Esterolone OR Norandrostenolone

Appendix 2

Outcome Definitions and Hierarchies

**Table 3:** Hierarchical Definitions and Measurement Priorities for Study Outcomes ND = nandrolone decanoate; DXA = dual-energy X-ray absorptiometry; SPA = single-photon absorptiometry; DPA = dual-photon absorptiometry; QCT = quantitative computed tomography; BMD = bone mineral density; BMC = bone mineral content; MRI = magnetic resonance imaging; CT = computed tomography; BIA = bioelectrical impedance analysis; 1RM = one-repetition maximum; MVIC = maximal voluntary isometric contraction; VAS = visual analogue scale; NPRS = numeric pain rating scale; ODI = Oswestry Disability Index; SF-36 = 36-item Short Form Health Survey; SF-12 = 12-item Short Form Health Survey; PCS = physical component summary; MCS = mental component summary; HAQ-DI = Health Assessment Questionnaire Disability Index; WOMAC = Western Ontario and McMaster Universities Osteoarthritis Index; SPPB = Short Physical Performance Battery; TUG = Timed Up and Go test; 6MWT = six-minute walk test; EQ-5D = EuroQol five-dimension questionnaire; QUALIOST = Quality of Life Questionnaire in Osteoporosis; OPAQ = Osteoporosis Assessment Questionnaire.

Outcome	Definition and Hierarchy
Primary Outcome	Incidence of clinical fractures, hierarchically prioritized as hip → vertebral → radial (forearm) fractures. This hierarchy reflects the clinical relevance of preventing major osteoporotic fractures in postmenopausal women treated with nandrolone decanoate (ND).
Secondary Outcome 1: Bone Mineral Density (BMD)	Preferred modality: DXA (areal, g/cm²). Anatomical priority for DXA measurements: Lumbar spine (L1-L4) → Femoral neck → Total hip → Trochanter → Ward’s triangle. Tie-breakers / alternative modalities (when DXA not available): A. Classical SPA/DPA: Distal radius (distal radius BMD / FMC); Lumbar spine L2-L4 (DPA/DXA). B. Other methods (e.g., QCT), as reported by individual trials.
Secondary Outcome 2: Bone Mineral Content (BMC)	Priority order (and measurement notes): Distal radius - fat-corrected BMC → Proximal radius (from the distal segment) - fat-corrected BMC → Distal radius - uncorrected BMC → Lumbar spine L2-L4 - BMC by DPA → Midshaft radius - BMC.
Secondary Outcome 3: Body Composition and Muscle Strength	Lean mass (preferred modality hierarchy): MRI/CT → DXA → bioelectrical impedance (BIA) → skinfolds. Muscle strength (preferred test hierarchy; lower-limb prioritized): Isokinetic dynamometry (peak torque, Nm) → Direct 1RM (e.g., leg press) → Estimated 1RM from multiple-RM tests → Maximal voluntary isometric contraction (MVIC) → Handgrip strength (only if no lower-limb measure available).
Secondary Outcome 4: Symptoms, Function, and Quality of Life	Pain: VAS → NPRS → ordinal scales. Function: ODI; SF-36 Physical Function / PCS; HAQ-DI; WOMAC (when applicable); SPPB; TUG; 6MWT. Quality of life: EQ-5D (index/utility); SF-36/SF-12 (PCS/MCS); osteoporosis-specific instruments (QUALIOST, OPAQ).
Secondary Outcome 5: Adverse Events	All adverse effects related to ND will be reported. When available, androgenic events (e.g., hirsutism, voice change, alopecia) will be specifically categorized.

Appendix 3

GRADE Methods and Explanatory Footnotes

**Table 4:** Summary of findings (GRADE) The risk in the intervention group (and its 95% CI) is based on the assumed risk in the comparator group and the relative effect of the intervention (and its 95% CI). Certainty ratings were assessed using the GRADE approach [32]. CI: confidence interval; RR: risk ratio; SMD: standardized mean difference; GRADE: Grading of Recommendations, Assessment, Development and Evaluation

Outcome	Participants (studies)	Relative effect (95% CI)	Absolute effects	Certainty (GRADE)	What happens
Fractures (RR)	74 (2 RCTs)	RR 0.37 (0.20–0.69)	Placebo: 51.5% • ND: 19.1% (10.3–35.5) → 32.4% fewer fractures	⨁⨁⨁◯ Moderate^a,b^	ND probably reduces fracture risk.
Bone Mineral Density (BMD) (SMD)	96 (3 RCTs)	–	SMD 0.31 higher (0.02–0.59)	⨁⨁◯◯ Low^c^	ND may slightly increase BMD.
Bone Mineral Content (BMC) (SMD)	96 (3 RCTs)	–	SMD 0.72 higher (0.34–1.10)	⨁⨁⨁◯ Moderate^d,e^	ND probably increases BMC.
Pain (SMD)	45 (2 RCTs)	–	SMD 0.76 lower (−1.36 to −0.15) (improvement)	⨁⨁⨁◯ Moderate^f,g^	ND probably reduces pain.
Muscle mass (SMD)	82 (2 RCTs)	–	SMD 0.74 higher (0.14–1.35)	⨁⨁⨁◯ Moderate^h,i^	ND probably increases lean mass.
Adverse events (RR)	234 (5 RCTs)	RR 4.59 (1.65–12.74)	Placebo: 3.5% • ND: 15.8% (6.0–76.9) → 12.3% more events	⨁⨁◯◯ Low^j,k^	ND may increase mild androgenic adverse events.

Explanations

a. Downgraded one level for risk of bias, due to limited reporting of randomization and allocation concealment in older trials.

b. Downgraded for imprecision (small sample and low number of fracture events).

c. Downgraded two levels for very serious risk of bias, due to multiple high-risk and “some concerns” judgments across key RoB 2 domains in the BMD trials-especially deviations from intended interventions, randomization issues, and incomplete reporting.

d. Downgraded one level for risk of bias (concerns in randomization, selective reporting, missing data).

e. Downgraded for imprecision, as the confidence interval spans from a small to a large effect size and the total sample size is below the optimal information size.

f. Downgraded one level for risk of bias, especially due to subjective outcome and limited blinding.

g. Downgraded for imprecision (small sample size).

h. Downgraded one level for risk of bias.

i. Downgraded one level for serious risk of bias (multiple high-risk domains) and one level for imprecision (small total sample).

j. Downgraded one level for risk of bias, mainly due to incomplete adverse-event reporting.

k. Downgraded for imprecision (wide CIs, few events in several trials).
